# Supplementary material for: An Eruption of LTR Retrotransposons in the Autopolyploid Genomes of Chrysanthemum nankingense (Asteraceae)
Source: Plants (Basel). 2022 Jan 25;11(3):315. doi: 10.3390/plants11030315 (PMC8839533; doi:10.3390/plants11030315)
Supplement: Supplementary file 1 [file plants-11-00315-s001.zip › TableS1.pdf]

Table S1. Codon neutral detection of Ty1RT based on the maximum likelihood method.

Codon	Triplet	Syn (s)	Nonsyn (n)	Syn sites (S)	Nonsyn sites (N)	dS	dN	dN-dS	*P*-value	Normalized dN-dS
1	ACA	18.00	0.00	1.00	2.00	18.00	0.00	-18.00	1.00	-5.19
2	GCG	4.00	0.00	1.00	2.00	4.00	0.00	-4.00	1.00	-1.15
3	TTC	10.00	0.00	0.37	2.63	26.98	0.00	-26.98	1.00	-7.78
4	CTA	18.00	4.00	1.26	1.69	14.29	2.37	-11.92	1.00	-3.44
5	CAC	3.00	1.00	0.37	2.63	8.09	0.38	-7.71	1.00	-2.23
6	GGA	5.00	0.00	1.00	1.87	5.00	0.00	-5.00	1.00	-1.44
7	CAA	0.50	4.50	0.36	2.49	1.38	1.81	0.43	0.69	0.12
8	CTG	6.00	0.00	1.15	1.85	5.23	0.00	-5.23	1.00	-1.51
9	AAA	1.00	4.00	0.32	2.39	3.16	1.68	-1.48	0.89	-0.43
10	GAA	0.00	3.00	0.29	2.44	0.00	1.23	1.23	0.71	0.35
11	ACG	5.00	10.00	0.57	2.24	8.85	4.47	-4.38	0.94	-1.26
12	GTT	1.00	3.00	0.36	2.13	2.80	1.41	-1.40	0.90	-0.40
13	TTT	1.50	3.50	0.64	2.36	2.33	1.48	-0.85	0.82	-0.25
14	GTC	3.00	3.00	0.37	2.53	8.09	1.19	-6.91	1.00	-1.99
15	AGC	1.67	2.33	0.33	2.37	5.08	0.98	-4.10	0.97	-1.18
16	CAA	6.00	2.00	0.92	2.08	6.52	0.96	-5.56	1.00	-1.60
17	CCC	0.00	4.00	0.27	2.42	0.00	1.66	1.66	0.66	0.48
18	GAA	3.00	3.00	0.88	1.96	3.40	1.53	-1.86	0.92	-0.54
19	GGA	4.25	2.75	0.43	2.51	9.97	1.09	-8.88	1.00	-2.56
20	TTC	2.00	4.00	0.99	2.01	2.03	1.99	-0.05	0.69	-0.01
21	GTT	2.00	2.00	0.58	2.42	3.43	0.83	-2.60	0.97	-0.75
22	GAT	6.00	11.00	0.73	2.16	8.20	5.09	-3.11	0.89	-0.90
23	CCA	5.50	3.50	0.69	2.16	8.01	1.62	-6.40	1.00	-1.85
24	GAG	1.50	8.50	0.41	2.37	3.67	3.59	-0.08	0.69	-0.02
25	TGC	3.50	5.50	0.76	2.13	4.61	2.59	-2.03	0.87	-0.58
26	TCG	0.00	4.00	0.35	2.58	0.00	1.55	1.55	0.60	0.45
27	AAC	2.00	4.00	0.36	2.39	5.61	1.67	-3.93	0.97	-1.13
28	CAT	6.00	2.00	0.88	2.12	6.83	0.94	-5.88	1.00	-1.70
29	GTC	2.00	2.00	0.37	2.08	5.40	0.96	-4.44	0.99	-1.28
30	TAC	1.33	2.67	0.39	2.31	3.41	1.15	-2.25	0.93	-0.65
31	AAA	5.00	2.00	0.95	1.97	5.25	1.02	-4.23	0.99	-1.22
32	CTG	2.00	6.00	0.60	2.22	3.34	2.70	-0.64	0.77	-0.18
33	AAC	1.50	2.50	0.41	2.29	3.67	1.09	-2.58	0.94	-0.74
34	AAA	2.50	6.50	0.88	2.05	2.83	3.17	0.34	0.59	0.10
35	GCT	7.50	3.50	0.89	2.06	8.40	1.70	-6.70	1.00	-1.93
36	TTG	1.00	1.00	0.49	2.00	2.04	0.50	-1.54	0.96	-0.44

37	TAT	4.00	5.00	0.99	1.98	4.03	2.52	-1.51	0.85	-0.43
38	GGT	3.00	4.00	0.74	2.06	4.03	1.94	-2.09	0.91	-0.60
39	CTC	1.33	2.67	0.38	2.30	3.54	1.16	-2.39	0.93	-0.69
40	AAA	2.00	2.00	0.48	2.19	4.16	0.91	-3.25	0.98	-0.94
41	CAA	5.00	4.00	0.91	1.94	5.50	2.06	-3.44	0.97	-0.99
42	GCC	2.00	1.00	0.88	2.05	2.28	0.49	-1.80	0.97	-0.52
43	CCT	5.50	2.50	1.00	1.86	5.49	1.35	-4.14	0.99	-1.20
44	CGG	2.00	4.00	0.82	1.98	2.44	2.02	-0.42	0.76	-0.12
45	GCA	0.50	3.50	0.24	2.25	2.11	1.56	-0.55	0.81	-0.16
46	TGG	4.00	2.00	0.37	2.12	10.79	0.94	-9.85	1.00	-2.84
47	TAC	1.00	4.00	0.38	2.51	2.61	1.59	-1.02	0.87	-0.29
48	GAT	3.00	3.00	0.55	2.15	5.46	1.40	-4.07	0.98	-1.17
49	AAG	5.00	5.00	0.86	2.07	5.83	2.41	-3.42	0.96	-0.99
50	CTA	2.00	3.00	0.74	2.02	2.71	1.48	-1.23	0.88	-0.35
51	TCA	5.00	5.00	0.69	2.27	7.22	2.21	-5.01	0.99	-1.45
52	TCT	4.00	2.00	0.37	2.61	10.81	0.77	-10.05	1.00	-2.90
53	TTC	2.00	3.00	0.75	2.25	2.67	1.33	-1.34	0.90	-0.39
54	CTC	3.83	4.17	0.71	2.22	5.37	1.88	-3.49	0.96	-1.01
55	ATT	5.50	8.50	0.67	2.21	8.20	3.84	-4.36	0.95	-1.26
56	GCT	2.00	3.00	0.49	2.51	4.08	1.20	-2.89	0.97	-0.83
57	AAT	1.00	4.00	0.68	2.32	1.47	1.72	0.25	0.68	0.07
58	AAT	2.00	2.00	0.40	2.58	5.06	0.78	-4.29	0.99	-1.24
59	TTC	2.00	5.00	0.71	2.22	2.83	2.26	-0.57	0.77	-0.16
60	ACC	0.00	2.00	0.40	2.28	0.00	0.88	0.88	0.73	0.25
61	AAA	3.00	4.00	0.83	2.15	3.63	1.86	-1.78	0.90	-0.51
62	GGT	2.00	5.00	0.88	1.89	2.27	2.65	0.37	0.61	0.11
63	TCG	3.00	3.00	0.65	2.16	4.65	1.39	-3.26	0.97	-0.94
64	GTT	2.00	2.00	0.41	2.50	4.87	0.80	-4.07	0.99	-1.18
65	GAC	2.00	2.00	0.69	2.12	2.90	0.94	-1.95	0.95	-0.56
66	CCA	3.00	4.00	0.79	2.12	3.81	1.89	-1.92	0.91	-0.55
67	ACT	4.50	5.50	0.81	2.09	5.54	2.63	-2.91	0.92	-0.84
68	CTT	5.00	2.00	0.37	2.42	13.49	0.83	-12.66	1.00	-3.65
69	TTT	1.50	5.50	0.55	2.36	2.72	2.33	-0.39	0.74	-0.11
70	ATA	1.00	1.00	0.28	2.34	3.61	0.43	-3.18	0.99	-0.92
71	CAA	1.00	6.00	0.47	2.17	2.12	2.77	0.65	0.64	0.19
72	TAT	1.00	8.00	0.57	2.39	1.76	3.34	1.58	0.46	0.46
73	CAC	3.00	7.00	0.95	1.98	3.16	3.53	0.37	0.59	0.11
74	GGG	2.50	6.50	0.94	1.97	2.66	3.30	0.64	0.54	0.18
75	GCA	4.50	7.50	0.70	2.13	6.41	3.53	-2.88	0.90	-0.83

76	CAT	4.83	4.17	0.86	1.99	5.65	2.09	-3.56	0.96	-1.03
77	ATC	3.00	3.00	0.82	1.94	3.68	1.55	-2.13	0.93	-0.62
78	TTA	1.00	3.00	0.66	2.26	1.51	1.33	-0.19	0.78	-0.05
79	ATT	1.00	6.00	0.68	2.08	1.47	2.89	1.41	0.45	0.41
80	GTT	4.00	2.00	0.62	2.12	6.42	0.94	-5.47	1.00	-1.58
81	CAA	4.00	2.00	0.83	2.12	4.81	0.94	-3.86	0.99	-1.11
82	ATT	2.00	6.00	0.45	2.10	4.43	2.86	-1.57	0.85	-0.45
83	TAC	18.50	4.50	0.73	2.12	25.24	2.13	-23.11	1.00	-6.67
84	GTG	6.00	3.00	0.58	2.42	10.30	1.24	-9.06	1.00	-2.61

NOTE. For each codon, estimates of the numbers of inferred synonymous (s) and nonsynonymous (n) substitutions are presented along with the numbers of sites that are estimated to be synonymous (S) and nonsyonymous (N). These estimates are produced using the joint Maximum Likelihood reconstructions of ancestral states under a Muse-Gaut model (Muse and Gaut 1994) of codon substitution and Felsenstein 1981 model (Felsenstein 1981) of nucleotide substitution. For estimating ML values, a tree topology was automatically computed. The test statistic dN - dS is used for detecting codons that have undergone positive selection, where dS is the number of synonymous substitutions per site (s/S) and dN is the number of nonsynonymous substitutions per site (n/N). A positive value for the test statistic indicates an overabundance of nonsynonymous substitutions. In this case, the probability of rejecting the null hypothesis of neutral evolution (*P*-value) is calculated (Pond and Frost 2005; Suzuki and Gojobori 1999). Values of *P* less than 0.05 are considered significant at a 5% level and are highlighted. Normalized dN - dS for the test statistic is obtained using the total number of substitutions in the tree (measured in expected substitutions per site). It is useful for making comparisons across data sets. Maximum Likelihood computations of dN and dS were conducted using HyPhy software package (Pond and Muse 2005). The analysis involved 45 nucleotide sequences. All positions containing gaps and missing data were eliminated. There were a total of 84 positions in the final dataset.
