# Supplementary material for: An Eruption of LTR Retrotransposons in the Autopolyploid Genomes of Chrysanthemum nankingense (Asteraceae)
Source: Plants (Basel). 2022 Jan 25;11(3):315. doi: 10.3390/plants11030315 (PMC8839533; doi:10.3390/plants11030315)
Supplement: Supplementary file 1 [file plants-11-00315-s001.zip › TableS2.pdf]

Table S2. Codon neutral detection of Ty3RT based on the maximum likelihood method

Codon	Triplet	Syn (s)	Nonsyn (n)	Syn sites (S)	Nonsyn sites (N)	dS	dN	dN-dS	*P*-value	Normalize dN-dS
1	AGG	11.00	0.00	0.76	2.22	14.54	0.00	-14.54	1.00	-5.22
2	ATG	0.00	0.00	0.00	3.00	0.00	0.00	0.00	0.00	0.00
3	TGC	10.00	0.00	0.36	2.28	27.79	0.00	-27.79	1.00	-9.98
4	GTG	9.00	0.00	1.00	2.00	9.00	0.00	-9.00	1.00	-3.23
5	GAC	3.00	0.00	0.36	2.64	8.34	0.00	-8.34	1.00	-3.00
6	TAT	0.00	0.00	0.36	2.00	0.00	0.00	0.00	0.00	0.00
7	CGA	11.00	0.00	1.06	1.73	10.40	0.00	-10.40	1.00	-3.74
8	GAA	4.50	4.50	0.36	2.33	12.35	1.93	-10.42	1.00	-3.75
9	TTG	4.00	2.00	0.76	1.94	5.29	1.03	-4.26	0.99	-1.53
10	AAC	3.00	0.00	0.36	2.64	8.34	0.00	-8.34	1.00	-3.00
11	AAG	4.50	3.50	0.38	2.24	11.95	1.56	-10.39	1.00	-3.73
12	TTA	5.00	5.00	0.92	2.03	5.41	2.47	-2.94	0.94	-1.06
13	ACG	6.00	0.00	1.00	2.00	6.00	0.00	-6.00	1.00	-2.16
14	GTG	3.50	3.50	0.84	2.16	4.19	1.62	-2.57	0.94	-0.92
15	AAG	3.50	2.50	0.35	2.25	10.05	1.11	-8.94	1.00	-3.21
16	AAT	3.00	1.00	0.36	2.64	8.34	0.38	-7.96	1.00	-2.86
17	CGT	5.00	4.00	0.77	2.06	6.46	1.94	-4.52	0.98	-1.62
18	TAC	5.00	4.00	0.36	2.25	13.90	1.78	-12.12	1.00	-4.35
19	CCA	4.00	0.00	1.00	2.00	4.00	0.00	-4.00	1.00	-1.44
20	CTC	10.00	2.00	0.89	2.10	11.25	0.95	-10.30	1.00	-3.70
21	CCC	8.00	1.00	1.00	2.00	7.98	0.50	-7.48	1.00	-2.69
22	AGA	9.00	5.00	0.70	2.15	12.8	2.33	-10.51	1.00	-3.78

						4				
23	ATT	3.00	0.00	0.72	2.28	4.15	0.00	-4.15	1.00	-1.49
24	GAT	5.00	1.00	0.35	2.57	14.23	0.39	-13.84	1.00	-4.97
25	GAC	4.00	2.00	0.35	2.52	11.38	0.79	-10.59	1.00	-3.81
26	TTG	9.00	0.00	0.90	1.78	9.95	0.00	-9.95	1.00	-3.57
27	TTT	4.00	2.00	0.48	2.42	8.28	0.83	-7.45	1.00	-2.68
28	GAT	1.00	2.00	0.36	2.64	2.78	0.76	-2.02	0.96	-0.73
29	CAG	4.00	2.00	0.32	2.33	12.36	0.86	-11.50	1.00	-4.13
30	TTG	11.00	3.00	0.82	1.93	13.35	1.56	-11.79	1.00	-4.24
31	CAA	1.00	3.00	0.34	2.43	2.93	1.23	-1.70	0.92	-0.61
32	GGT	4.00	0.00	1.00	1.99	4.00	0.00	-4.00	1.00	-1.44
33	TCT	9.00	3.00	1.00	1.80	9.00	1.67	-7.33	1.00	-2.63
34	AGT	3.00	5.00	0.38	2.44	7.94	2.05	-5.90	0.99	-2.12
35	ATC	5.00	4.00	0.92	2.07	5.42	1.93	-3.49	0.97	-1.25
36	TAT	4.00	3.00	0.36	2.35	11.12	1.28	-9.84	1.00	-3.53
37	TCC	5.50	2.50	0.97	1.63	5.66	1.53	-4.13	0.98	-1.48
38	AAG	5.00	0.00	0.33	2.25	15.09	0.00	-15.09	1.00	-5.42
39	ATT	9.50	2.50	0.83	2.16	11.49	1.16	-10.33	1.00	-3.71
40	GAT	7.00	1.00	0.36	2.64	19.45	0.38	-19.07	1.00	-6.85
41	CTC	6.00	2.00	0.75	1.85	8.02	1.08	-6.94	1.00	-2.49
42	CGG	5.50	3.50	0.63	2.22	8.76	1.57	-7.19	1.00	-2.58
43	TCG	7.00	0.00	1.00	1.93	7.00	0.00	-7.00	1.00	-2.51
44	GGT	5.00	0.00	1.00	1.73	5.00	0.00	-5.00	1.00	-1.80
45	TAT	3.00	1.00	0.36	2.01	8.34	0.50	-7.84	1.00	-2.82
46	CAT	3.00	0.00	0.36	2.64	8.34	0.00	-8.34	1.00	-3.00
47	CAG	4.00	0.00	0.32	2.32	12.4	0.00	-12.43	1.00	-4.47

						3				
48	CTT	7.00	1.00	1.03	1.92	6.77	0.52	-6.25	1.00	-2.24
49	CGA	7.50	1.50	0.93	1.74	8.06	0.86	-7.20	1.00	-2.59
50	GTT	4.00	2.00	0.76	2.24	5.28	0.89	-4.39	1.00	-1.58
51	CGG	8.50	7.50	1.13	1.81	7.51	4.15	-3.36	0.92	-1.21
52	GAA	1.00	4.00	0.33	2.41	3.04	1.66	-1.38	0.89	-0.50
53	GAG	2.00	4.00	0.34	2.36	5.97	1.70	-4.27	0.97	-1.53
54	GAT	6.00	1.00	0.36	2.64	16.6	0.38	-16.30	1.00	-5.85
						7				
55	ATT	6.00	3.00	0.83	2.17	7.21	1.38	-5.83	1.00	-2.09
56	CCG	4.00	4.00	0.75	2.24	5.31	1.79	-3.52	0.97	-1.27
57	AAA	5.00	0.00	0.33	2.26	15.3	0.00	-15.37	1.00	-5.52
						7				
58	ACA	5.00	0.00	1.00	2.00	5.00	0.00	-5.00	1.00	-1.80
59	GCG	5.00	1.00	1.00	2.00	5.00	0.50	-4.50	1.00	-1.62
60	TTT	2.00	0.00	0.36	2.64	5.56	0.00	-5.56	1.00	-2.00
61	AGA	3.00	3.00	0.66	1.96	4.55	1.53	-3.02	0.96	-1.08
62	ACA	4.00	0.00	1.00	2.00	4.00	0.00	-4.00	1.00	-1.44
63	CGT	6.00	5.00	0.89	2.00	6.76	2.50	-4.27	0.98	-1.53
64	TAC	6.00	3.00	0.35	2.17	17.0	1.38	-15.69	1.00	-5.64
						7				
65	GGT	5.00	1.00	1.00	1.92	5.01	0.52	-4.49	1.00	-1.61
66	CAT	3.00	0.00	0.36	2.64	8.34	0.00	-8.34	1.00	-3.00
67	TAC	5.00	0.00	0.36	2.00	13.9	0.00	-13.90	1.00	-4.99
						0				
68	GAG	3.00	0.00	0.33	2.34	9.09	0.00	-9.09	1.00	-3.27
69	TTT	6.00	1.00	0.36	2.64	16.6	0.38	-16.27	1.00	-5.85
						5				
70	CAA	6.00	3.00	0.78	2.01	7.71	1.49	-6.22	1.00	-2.23
71	GTG	5.00	1.00	1.00	2.00	5.00	0.50	-4.50	1.00	-1.62
72	ATG	0.00	0.00	0.00	3.00	0.00	0.00	0.00	0.00	0.00
73	CCG	7.00	1.00	1.00	2.00	7.00	0.50	-6.50	1.00	-2.34
74	TTT	4.00	0.00	0.36	2.64	11.1	0.00	-11.12	1.00	-3.99
						2				

75	GGA	6.00	0.00	1.00	1.79	6.00	0.00	-6.00	1.00	-2.16
76	TTG	5.00	2.00	0.72	1.90	6.91	1.05	-5.85	1.00	-2.10
77	ACT	6.00	1.00	1.00	2.00	6.00	0.50	-5.50	1.00	-1.98
78	AAT	4.00	0.00	0.36	2.64	11.12	0.00	-11.12	1.00	-3.99
79	GCA	7.00	0.00	1.00	2.00	7.00	0.00	-7.00	1.00	-2.51
80	CCT	5.00	1.00	1.00	2.00	4.99	0.50	-4.49	1.00	-1.61
81	GCG	7.00	1.00	1.00	1.98	7.00	0.50	-6.50	1.00	-2.33
82	GTA	5.00	4.00	0.99	2.01	5.05	1.99	-3.06	0.96	-1.10
83	TTC	1.00	0.00	0.36	2.64	2.78	0.00	-2.78	1.00	-1.00
84	ATG	2.00	2.00	0.14	2.72	14.14	0.74	-13.40	1.00	-4.82
85	GAT	4.00	3.00	0.61	2.36	6.60	1.27	-5.33	0.99	-1.91
86	CTC	5.00	3.00	1.01	1.97	4.93	1.52	-3.40	0.98	-1.22
87	ATG	0.00	0.00	0.00	3.00	0.00	0.00	0.00	0.00	0.00
88	AAT	1.00	0.00	0.36	2.64	2.78	0.00	-2.78	1.00	-1.00
89	AGA	6.00	4.00	0.91	1.80	6.56	2.22	-4.34	0.98	-1.56
90	GTT	6.00	1.00	1.01	1.99	5.96	0.50	-5.46	1.00	-1.96
91	TGT	3.00	1.00	0.36	2.45	8.34	0.41	-7.93	1.00	-2.85
92	AAA	3.00	10.00	0.41	2.27	7.29	4.41	-2.88	0.87	-1.03
93	CCG	4.00	6.00	0.95	2.04	4.23	2.94	-1.29	0.82	-0.46
94	TAT	3.50	6.50	0.38	2.07	9.10	3.15	-5.95	0.97	-2.14
95	TTG	13.00	0.00	1.14	1.75	11.43	0.00	-11.43	1.00	-4.11
96	GAC	4.00	3.00	0.52	2.47	7.76	1.21	-6.55	1.00	-2.35
97	AAG	4.00	2.00	0.32	2.28	12.32	0.88	-11.44	1.00	-4.11
98	TTT	2.50	3.50	0.40	2.56	6.23	1.37	-4.87	0.98	-1.75
99	GTG	5.00	0.00	1.00	2.00	5.00	0.00	-5.00	1.00	-1.80
100	ATA	7.00	3.00	0.84	2.16	8.36	1.39	-6.97	1.00	-2.51
101	GTG	6.00	0.00	1.00	2.00	6.00	0.00	-6.00	1.00	-2.16
102	TTC	4.00	0.00	0.36	2.64	11.12	0.00	-11.12	1.00	-3.99
103	ATC	3.00	1.00	0.58	2.42	5.14	0.41	-4.72	1.00	-1.70

104	GAC	3.00	1.00	0.36	2.63	8.37	0.38	-7.99	1.00	-2.87
105	GAC	5.00	0.00	0.36	2.64	13.90	0.00	-13.90	1.00	-4.99
106	ATC	3.00	0.00	0.72	2.28	4.16	0.00	-4.16	1.00	-1.50
107	TTG	8.00	0.00	1.26	1.70	6.36	0.00	-6.36	1.00	-2.29
108	ATT	4.00	3.00	0.77	2.23	5.17	1.35	-3.82	0.99	-1.37
109	TAT	4.00	1.00	0.36	2.02	11.12	0.50	-10.62	1.00	-3.82
110	TCA	4.00	2.00	0.75	2.23	5.31	0.90	-4.41	1.00	-1.58
111	AAG	2.50	8.50	0.36	2.30	6.98	3.70	-3.28	0.89	-1.18
112	AGC	3.00	8.00	0.72	2.23	4.15	3.58	-0.57	0.73	-0.21
113	AAA	4.33	10.67	0.48	2.37	8.97	4.49	-4.48	0.93	-1.61
114	GAA	2.67	8.33	0.37	2.31	7.17	3.60	-3.56	0.90	-1.28
115	GAG	2.00	4.00	0.36	2.35	5.60	1.70	-3.90	0.97	-1.40
116	CAT	3.00	0.00	0.36	2.64	8.34	0.00	-8.34	1.00	-3.00
117	GAA	5.50	7.50	0.47	2.27	11.58	3.30	-8.28	0.99	-2.97
118	GAG	5.00	8.00	0.41	2.29	12.09	3.49	-8.60	0.99	-3.09
119	CAT	5.00	3.00	0.36	2.62	13.84	1.14	-12.69	1.00	-4.56
120	TTA	4.00	3.00	0.78	1.95	5.10	1.54	-3.56	0.98	-1.28
121	AGG	4.00	7.00	0.73	2.17	5.51	3.23	-2.28	0.88	-0.82
122	ACG	8.25	11.75	0.79	2.07	10.40	5.68	-4.72	0.94	-1.70
123	ATT	5.00	1.00	0.91	2.09	5.50	0.48	-5.02	1.00	-1.80
124	TTA	9.00	3.00	0.77	1.92	11.72	1.56	-10.16	1.00	-3.65
125	AGG	2.00	9.00	0.35	2.35	5.76	3.83	-1.93	0.84	-0.69
126	CTA	6.50	6.50	0.79	1.99	8.23	3.26	-4.97	0.97	-1.79
127	CTC	9.00	2.00	0.80	2.13	11.23	0.94	-10.29	1.00	-3.70
128	AAG	3.00	5.00	0.50	2.28	5.95	2.20	-3.76	0.96	-1.35
129	AAT	3.00	12.00	0.41	2.28	7.36	5.26	-2.09	0.82	-0.75

130	GAG	6.00	5.00	0.35	2.45	17.26	2.04	-15.21	1.00	-5.47
131	AAG	6.00	7.00	0.34	2.31	17.83	3.04	-14.80	1.00	-5.32
132	TTG	5.00	2.00	0.82	1.92	6.09	1.04	-5.05	1.00	-1.81
133	TAT	3.00	2.00	0.36	2.13	8.34	0.94	-7.40	1.00	-2.66
134	GCC	7.00	1.00	1.00	2.00	7.00	0.50	-6.50	1.00	-2.34
135	AAG	5.00	0.00	0.33	2.25	14.99	0.00	-14.99	1.00	-5.39
136	TTC	5.17	5.83	0.58	2.41	8.92	2.42	-6.50	0.99	-2.34
137	TCG	7.00	3.00	0.85	1.91	8.20	1.57	-6.63	1.00	-2.38
138	AAG	0.00	0.00	0.34	2.25	0.00	0.00	0.00	0.00	0.00
139	TGT	0.00	0.00	0.36	2.28	0.00	0.00	0.00	0.00	0.00
140	GTT	8.00	5.00	0.99	2.01	8.10	2.49	-5.61	0.99	-2.01
141	TTC	3.00	0.00	0.36	2.64	8.34	0.00	-8.34	1.00	-3.00
142	GGG	0.00	9.00	0.89	2.04	0.00	4.42	4.42	0.04	1.59
143	TGT	0.50	12.50	0.36	2.33	1.38	5.36	3.99	0.31	1.43

NOTE. For each codon, estimates of the numbers of inferred synonymous (s) and nonsynonymous (n) substitutions are presented along with the numbers of sites that are estimated to be synonymous (S) and nonsyonymous (N). These estimates are produced using the joint Maximum Likelihood reconstructions of ancestral states under a Muse-Gaut model (Muse and Gaut 1994) of codon substitution and Felsenstein 1981 model (Felsenstein 1981) of nucleotide substitution. For estimating ML values, a tree topology was automatically computed. The test statistic dN - dS is used for detecting codons that have undergone positive selection, where dS is the number of synonymous substitutions per site (s/S) and dN is the number of nonsynonymous substitutions per site (n/N). A positive value for the test statistic indicates an overabundance of nonsynonymous substitutions. In this case, the probability of rejecting the null hypothesis of neutral evolution (*P*-value) is calculated (Pond and Frost 2005; Suzuki and Gojobori 1999). Values of *P* less than 0.05 are considered significant at a 5% level and are highlighted. Normalized dN - dS for the test statistic is obtained using the total number of substitutions in the tree (measured in expected substitutions per site). It is useful for making comparisons across data sets. Maximum Likelihood computations of dN and dS were conducted using HyPhy software package (Pond and Muse 2005). The analysis involved 36 nucleotide sequences. All positions containing gaps and missing data were eliminated. There were a total of 143 positions in the final dataset
