# Supplementary material for: An Eruption of LTR Retrotransposons in the Autopolyploid Genomes of Chrysanthemum nankingense (Asteraceae)
Source: Plants (Basel). 2022 Jan 25;11(3):315. doi: 10.3390/plants11030315 (PMC8839533; doi:10.3390/plants11030315)
Supplement: Supplementary file 1 [file plants-11-00315-s001.zip › TableS3.pdf]

Table S3. Adaptor and primer sequences used for AFLP and SSAP analysis.

Adaptors/primers	Sequence (5'–3')
***Mse*I adaptor-1**	GACGATGAGTCCTGAG
***Mse*I adaptor-2**	TACTCAGGACTCAT
***EcoR*I adaptor-1**	CTCGTAGACTGCGTACC
***EcoR*I adaptor-2**	AATTGGTACGCAGTCTAC
***EcoR*I pre-selective primer**	GACTGCGTACCAATTCA
***Mse*I pre-selective primer**	GATGAGTCCTGAGTAAC
***EcoR*I selective primer-2**	GACTGCGTACCAATTCAAG
***EcoR*I selective primer-3**	GACTGCGTACCAATTCACA
***EcoR*I selective primer-4**	GACTGCGTACCAATTCACT
***EcoR*I selective primer-6**	GACTGCGTACCAATTCACG
***EcoR*I selective primer-7**	GACTGCGTACCAATTCAGC
***EcoR*I selective primer-8**	GACTGCGTACCAATTCAGG
***Mse*I selective primer-2**	GATGAGTCCTGAGTAACAC
***Mse*I selective primer-3**	GATGAGTCCTGAGTAACAG
***Mse*I selective primer-4**	GATGAGTCCTGAGTAACAT
***Mse*I selective primer-5**	GATGAGTCCTGAGTAACTA
***Mse*I selective primer-6**	GATGAGTCCTGAGTAACTC
***Mse*I selective primer-7**	GATGAGTCCTGAGTAACTG
***Mse*I selective primer-8**	GATGAGTCCTGAGTAACTT
