# Supplementary figures and images for: An Eruption of LTR Retrotransposons in the Autopolyploid Genomes of Chrysanthemum nankingense (Asteraceae)
Source: Plants (Basel). 2022 Jan 25;11(3):315. doi: 10.3390/plants11030315 (PMC8839533; doi:10.3390/plants11030315)

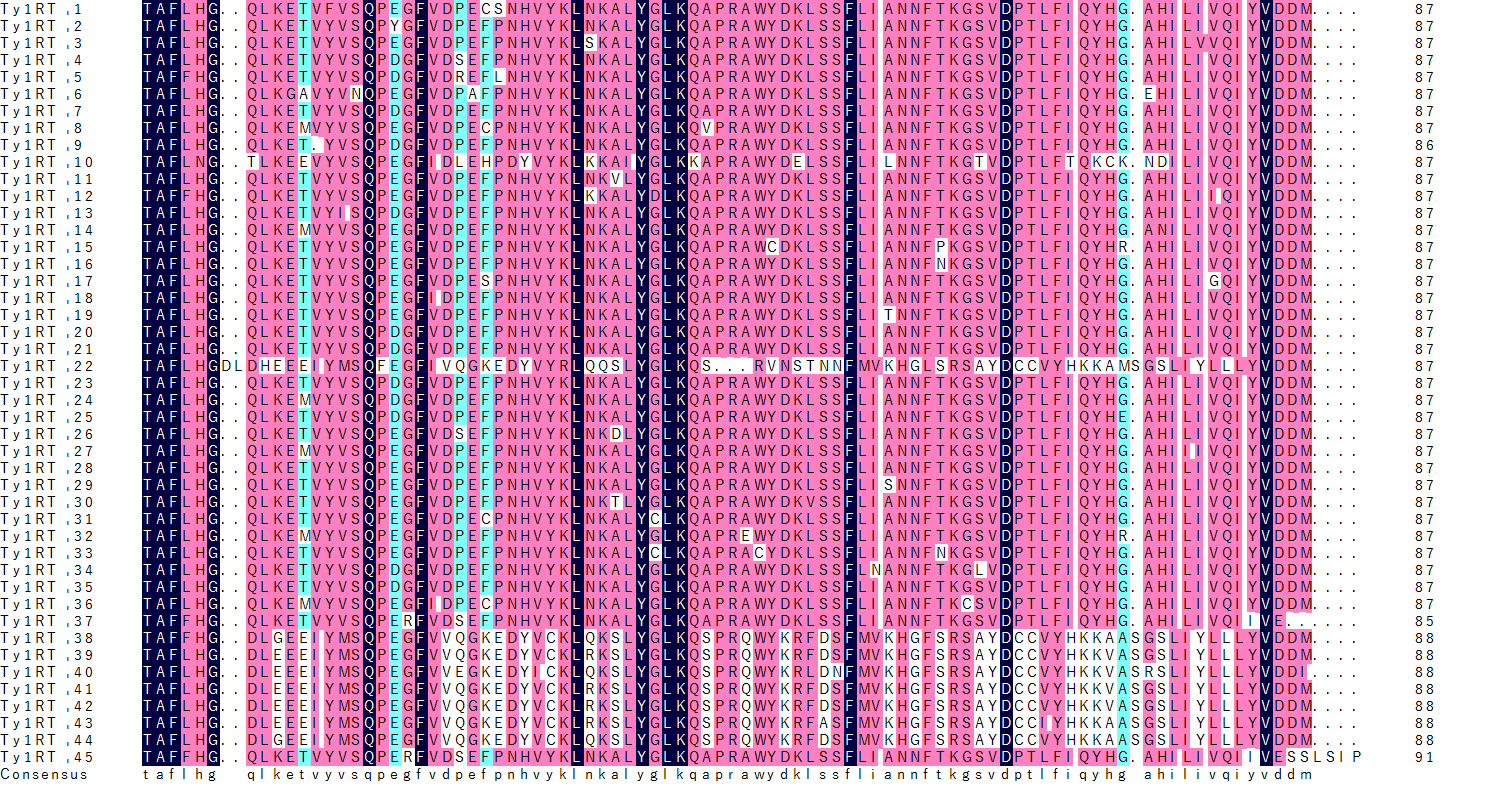

Supplement: Supplementary file 1 [file plants-11-00315-s001.zip › Fig.S1.tif]

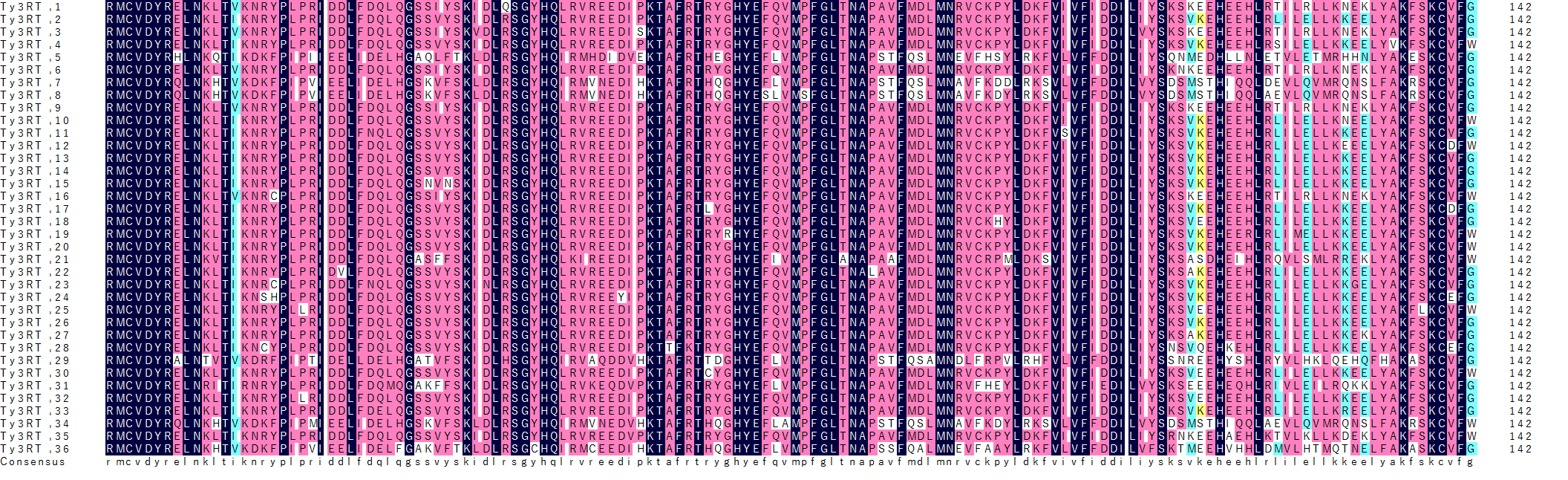

Supplement: Supplementary file 1 [file plants-11-00315-s001.zip › Fig.S2.tif]

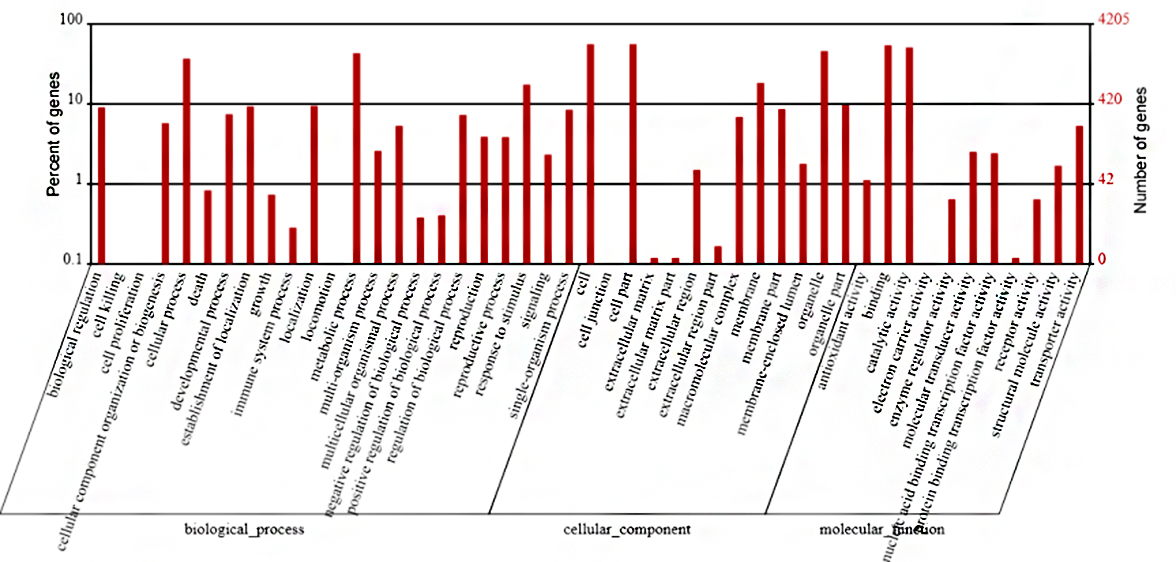

Supplement: Supplementary file 1 [file plants-11-00315-s001.zip › Fig.S3.tif]
